# Birth Season and Breed Effects on Newborn *Longissimus Thoracis* and *Semimembranosus* Muscles: Insights from the Nero Di Lomellina Piglets

**DOI:** 10.3390/ani16040655

**Published:** 2026-02-18

**Authors:** Margherita Pallaoro, Giorgio Mirra, Lucia Aidos, Mirko Sergio, Mauro Di Giancamillo, Raffaella Rossi, Annamaria Costa, Eleonora Buoio, Silvia Michela Mazzola, Silvia Clotilde Modina, Alessia Di Giancamillo

**Affiliations:** 1 Department of Biomedical Sciences for Health (SCIBIS), University of Milan (UNIMI), Via Mangiagalli 31, 20133 Milano, MI, Italy; giorgio.mirra@unimi.it; 2 Department of Veterinary Medicine and Animal Sciences (DIVAS), University of Milan (UNIMI), Via dell’Università 6, 26900 Lodi, LO, Italy; lucia.aidos@unimi.it (L.A.); mirko.sergio@unimi.it (M.S.); mauro.digiancamillo@unimi.it (M.D.G.); raffaella.rossi@unimi.it (R.R.); annamaria.costa@unimi.it (A.C.); eleonora.buoio@unimi.it (E.B.); silvia.mazzola@unimi.it (S.M.M.); silvia.modina@unimi.it (S.C.M.)

**Keywords:** pig breed, birth season, muscle, MRFs, HSPs, CSPs

## Abstract

*Background*: Understanding the factors influencing muscle development is essential for the livestock industry. This study aims to evaluate the effect of birth season and breed on the muscles *Longissimus thoracis* (LT) and *Semimembranosus* (SM) from newborn piglets of the local Italian breed Nero di Lomellina (NL) and the Commercial Hybrid Large WhitexDuroc (CH), born in winter (W) and summer (S). *Methods*: Muscles’ morphological features were evaluated, and the expression of Myogenic Regulatory Factors (MRFs: MYF5, MYOD, MYOG, MYF6) and heat and cold shock proteins (HSP27, HSP70, HSP90, CIRBP, RMB3) was assessed by quantitative PCR. *Results*: Both muscles showed a larger fiber cross-sectional area (LT: NL/S > NL/W, *p* = 0.035. SM: NL/S > NL/W, *p* = 0.035; CH/S > CH/W, *p* = 0.05) and a lower total number of fibers in summer piglets (LT: NL/S < NL/W, *p* = 0.05. SM: NL/S < NL/W, *p* = 0.033). In LT, MYF6 was higher, mainly in NL, in S (NL/S > NL/W, *p* < 0.0001; NL/S > CH/S, *p* = 0.0002), as well as HSP27 (NL/S > NL/W, *p* = 0.0001; NL/S > CH/S, *p* = 0.0018), HSP70 (NL/S > NL/W, *p* = 0.044. CH/S > CH/W, *p* = 0.0018), HSP90 (NL/S > NL/W, *p* < 0.0001; NL/S > CH/S, *p* = 0.023; CH/S > CH/W, *p* = 0.027), CIRBP NL/S > NL/W, *p* = 0.003; CH/S > CH/W, *p* = 0.0008), and RBM3 (NL/S > NL/W, *p* = 0.01; NL/S > CH/S, *p* = 0.036). In SM, MYF5 was higher in W in both breeds (NL/W > NL/S, *p* = 0.008; CH/W > CH/S, *p* < 0.0001; CH/W > NL/S, *p* = 0.012). Similarly, MYOD (NL/W > NL/S, *p* = 0.045), MYOG (NL/W > NL/S, *p* = 0.002; CH/W > CH/S, *p* = 0.025), and CIRBP (NL/W > NL/S, *p* = 0.003; NL/W > CH/W, *p* = 0.004) were mainly expressed in winter, while HSP90 was expressed in summer in CH. *Conclusions*: Our results demonstrate that muscle development in piglets at birth can vary between breeds and along different birth seasons, with an enhanced development observed mainly in summer newborns. These results could be helpful for improvement programs for NL and other local breeds by linking muscle development at birth to seasonal adaptation.

## Simple Summary

Muscle development and growth can be influenced by several factors. This study investigated how the season of birth (winter or summer) and the breed affect muscle development in newborn piglets. We compared a local Italian pig breed, Nero di Lomellina, with a widely used commercial crossbreed. We considered the muscles *Longissimus thoracis* and *Semimembranosus* and assessed their morphology and the expression of genes related to muscle growth and stress response. Piglets born in summer showed more developed muscle tissue, especially in the local breed. These findings suggest that muscle development at birth may vary between seasons of birth and breeds. This information could be useful to promote and improve local pig breeds; besides, understanding how early muscle growth is affected by season can support more sustainable and effective breeding strategies in traditional farming systems.

## 1. Introduction

The development of skeletal muscles and the establishment of a correct musculoskeletal phenotype in pigs are crucial for pig breeding and meat production. Skeletal muscle is a heterogeneous tissue consisting of mononucleated satellite cells, which serve as muscle stem niches, and differentiated multinucleated fibers, besides connective, adipose, endothelial, and nervous tissues [1,2]. During embryonic development, myogenic cells fuse, forming primary fibers between day 35 and day 60 of gestation. Subsequently, during fetal development, between days 55 and 90 of gestation, secondary muscle fibers form [3]. These two waves of fiber development determine the number of muscle fibers, which appears to be mostly fixed at birth [4]. Postnatally, muscle growth occurs primarily through the hypertrophic growth of existing muscle fibers; additionally, satellite cells and their progeny play a crucial role in muscle development, growth, and regeneration [5].

The development of skeletal muscles is under the control of Myogenic Regulatory Factors (MRFs) [6,7,8], while the protein Paired Box 7 (PAX7) guarantees tissue homeostasis between differentiating cells and the muscle stem niche by regulating the maintenance and renewal of satellite cells [9]. MRFs are basic helix-loop-helix transcription factors that can be divided into early and late myogenic factors. The early ones are Myogenic factor 5 (MYF5) and Myoblast Determination protein 1 (MYOD), while the late ones are Myogenin (MYOG) and Myogenic factor 6 (MYF6) [10]. MYF5 is the earliest expressed factor, and it acts synergistically with MYOD to commit myogenic precursor cells toward the skeletal myoblast lineage and to determine myoblasts [11]. MYOG regulates the final differentiation of myoblasts and their fusion into multinucleated fibers [12]. Lastly, MYF6 is associated with the muscle fibers’ maturation and differentiation process [13]. The expression pattern of MRFs drives myoblast proliferation, differentiation, and fiber hypertrophy [14].

The perinatal expression of these targets and the development of muscles can be affected by the genetic background of the animal, as in chickens [15], cattle [16], and different breeds of pigs [17], finally leading to various qualities and properties of meat [18,19,20]. Genetic selection reduces pigs’ ability to cope with several stressors that can alter animal development [21]. Environmental factors can impact muscle growth by inducing the expression of the chaperone heat shock proteins (HSPs). Most studies on the impact of HSPs on myogenesis were performed in vitro on myoblasts of different species, such as pigs [22,23], humans [24], and cattle [25], under different temperatures and oxygen tensions. The main HSPs identified in the chaperone mechanisms were HSP27, HSP70, and HSP90 [22,26]. In addition, a new class of temperature-related chaperone molecules has been recently identified; they are called cold shock proteins (CSPs) since they were first identified in microorganisms under acute cold stress. Cold-inducible RNA-binding protein (CIRBP) and RNA-binding motif protein (RBM3) are two evolutionarily conserved RNA-binding proteins that are transcriptionally upregulated in response to low temperature and regulate post-transcriptional modification in the myogenic program [27]. Although their function in bacteria is widely studied, their role in mammals requires further clarification [28].

Research has focused on pig muscle growth to develop new strategies for the livestock industry. This involves promoting the farming of breeds that are adaptable to diverse climatic and breeding conditions, thereby improving productivity while ensuring sustainability across different environments. [29,30]. Most studies focus on the *Longissimus thoracis* (LT) muscle for its implication in fresh meat production; however, the *Semimembranosus* (SM) muscle, as well, must be taken into consideration for its involvement in cooked ham production [31]. Moreover, the two muscles have different functions: LT contributes mainly to posture maintenance, while SM is involved in locomotion. These functional differences may lead to distinct microscopic and molecular features.

Therefore, the aim of the present study is to investigate how birth season and breed affect the characteristics of LT and SM muscles in newborn piglets. The research includes piglets from the local Italian breed Nero di Lomellina (NL) and the Commercial Hybrid Large White x Duroc (CH), born either during winter (W) or during summer (S).

## 2. Materials and Methods

### 2.1. Experimental Design

The trial was conducted on a commercial farm in northern Italy where NL and CH pigs are all reared under the same conditions and by the EU and Italian guidelines (2010/63/EU; D. Lgs. n. 26/2014) (European Commission 2020). The sows of two genetic types were selected on the basis of parity (2.9 ± 0.15 CH vs. 3.2 ± 0.34 NL; *p* = 0.35). As expected, the litter sizes differ between the two genetic types, with a higher total number of born piglets (13.5 ± 0.24 CH vs. 12.00 ± 0.65 NL; *p* = 0.01) and a higher number of born alive piglets (11.3 ± 0.33 CH vs. 9.7 ± 0.57 NL; *p* = 0.03) in CH than in NL. Samples of LT and SM muscles were collected from NL and CH newborn piglets born in two different birth seasons (W: winter, animals born in December, average environmental temperature 2.8 °C; S: summer, animals born in September, average environmental temperature 20.9 °C), so that four experimental groups were formed, considering the genetics and the season of birth: NL/S, NL/W, CH/S, and CH/W (number of animals per experimental group = 7; each piglet was obtained from a distinct litter). No animal was sacrificed for experimental purposes: the piglets were born alive and died crushed under the sow right after birth (4–6 h, females, wean weight 1.3 ± 0.05 kg) [32]. The experimental design was approved in all its parts by the Animal Welfare Committee of Università degli Studi di Milano (OPBA_89_2021).

### 2.2. Evaluation of Piglets and Muscles Weight

Piglets were individually weighed, and muscles were collected, weighed, measured, and processed for the analyses.

### 2.3. Muscle Sampling

After evaluating the weight and the cross-sectional area of each muscle, 1 cm^3^ biopsies of LT and SM were excised and partly frozen in liquid nitrogen for histological evaluation and partly stored in RNA Later (QIAGEN, Hilden, Germany) at −80 °C for gene expression analyses. For the freezing procedure, muscle biopsies were embedded in Killik O.C.T. embedding medium (Bio-Optica, Milan, Italy) and frozen in −80 °C isopentane, previously cooled in liquid nitrogen. Histological samples were finally stored at −20 °C [33].

### 2.4. Morphological and Histometric Evaluation—Hematoxylin–Eosin Staining

To assess structural details, LT and SM morphology were evaluated by Hematoxylin–Eosin (HE) staining on 7 µm cryosections. Briefly, sections were cut at the cryostat (MICROM HM 505E, Walldorf, Germany) and left to air dry for five minutes [33]. After a two-minute rinse in distilled water, nuclei were stained for 10 min in Mayer’s Hematoxylin (Sigma-Aldrich, Milan, Italy), while the cytoplasm was stained in Eosin (Bio-Optica, Milan, Italy) for 90 s. Sections were finally dehydrated by an Ethanol-Xylene ascending concentration scale and mounted in a DPX mounting medium (Sigma-Aldrich, Milan, Italy). Images were acquired using an optical microscope (Optika B-1000, 6 Vdc 2.5A—XLED8, Optika S.r.l., Ponteranica, Italy).

Histometric measurements were performed on transversal fibers. The evaluation of transversal fibers’ cross-sectional area (CSA) and density (number of fibers/mm^2^) was performed on 40× magnification images by Optika Proview software (64-bit, v4.11.18081.20201205; Optika S.r.l., Ponteranica, Italy). Specifically, 300 transversal fibers were randomly selected for each muscle within 5 acquired fields/animal/season to evaluate fibers’ CSA. Fiber density was assessed in 5 acquired fields/animal/season: the number of muscle fibers in the defined counting area (11,150 um^2^) was normalized from µm^2^ to mm^2^ to obtain n° muscle fibers/mm^2^. Finally, the total number of fibers for each muscle was calculated by multiplying the fibers’ density by the muscle’s cross-sectional area [34]. Analyses were performed blind to avoid observer bias.

### 2.5. Gene Expression Evaluation—MRFs, HSPs, CSPs

The expression of MRFs, HSPs, and CSPs was studied by real-time PCR (BioRad iQ5 Real-Time PCR System, v2.1.97.1001; Bio-Rad Laboratories, Hercules, CA, USA). 30 mg of each sample was used for RNA extraction by RNeasy Mini Kit (QIAGEN, Hilden, Germany). RNA concentration was assessed at the Nanodrop (Thermo Scientific, Waltham, MA, USA), and RNA quality was verified by electrophoresis on a 1% agarose gel, confirming intact RNA. cDNA was synthesized from 1000 ng of RNA per sample using the QuantiTect Rev. Transcription Kit (QIAGEN, Hilden, Germany). After checking reverse transcription by qualitative PCR for each sample and against each target, cDNA samples were used to assess targets’ expression in real-time PCR. The reaction mixture for real-time PCR was prepared with SsoAdvanced Universal SYBR Green Supermix (Bio-Rad, Hercules, CA, USA) at a final concentration of 1×, primers at a final concentration of 0.5 µM, and nuclease-free water. For each sample, the expression of every gene was compared to the average calibrator sample, and the relative expression values were calculated as ΔΔCt measure by using two housekeeping genes as references, as already performed in previous studies of our group [34,35].

The chosen housekeeping genes were *DRAP1* and *BETA ACTIN* [36]. After a proper assessment, the thermal cycling steps for each target were chosen: 95 °C 3′ + (95 °C 20″ + 59 °C 60″) × 40 + 55 °C 60″ + 95 °C up to temperature for *PAX7*, *MYOG*, *MYOD*, *BETA ACTIN*, *HSP90* and *CIRBP*; 95 °C 3′ + (95 °C 20″ + 56 °C 20″ + 72 °C 40″) × 40 + 55 °C 60″ + 95 °C up to temperature for *MYF5*, *MYF6*, *DRAP1* and *RBM3*; 95 °C 3′ + (95 °C 20″ + 60 °C 40″) × 40 + 55 °C 60″ + 95 °C up to temperature for *HSP70*; 95 °C 3′ + (95 °C 20″ + 59 °C 40″) × 40 + 55 °C 60″ + 95 °C up to temperature for *HSP27*.

Table 1 shows the sequence of the chosen primers.

### 2.6. Statistical Analysis

Statistical analysis was performed by PRISM—GraphPad by Dotmatics (GraphPad Prism 9.5.1.733, CA7F06FF8BF). Data were analyzed by a 2-way ANOVA test with birth group (W and S) and breeds (NL and CH) as the main factors. The effect of the individual factors is presented only when significant for datasets where the interaction between breed and birth season did not show statistical significance. Data are presented as mean ± standard error of the mean. Differences between means were considered significant at *p* < 0.05 (*), *p* < 0.01 (**), *p* < 0.001 (***), and *p* < 0.0001 (****).

## 3. Results

For clearer reading, morphological and molecular results are presented separately for each muscle.

### 3.1. Evaluation of Piglets and Muscle Weight

The collected piglets were homogeneous for weight at birth: indeed, no differences were found between breeds and between birth seasons (NL/W 1.22 ± 0.06 vs. NL/S 1.34 ± 0.14 kg, *p* = 0.58; CH/W 1.33 ± 0.06 kg vs. CH/S 1.4 ± 0.1 kg, *p* = 0.6; NL/W vs. CH/W, *p* = 0.53; NL/S vs. CH/S, *p* = 0.71). The weight of the LT muscle was lower in NL/S than in CH/S (4.24 ± 1.18 g vs. 10.39 ± 1.07 g, *p* = 0.022); no differences were found between NL and CH for winter birth (5.62 ± 0.27 vs. 10.26 ± 1.28 g, *p* = 0.42) and between the two birth seasons for NL (5.62 ± 0.27 g vs. 4.24 ± 1.18 g, *p* = 0.37) and CH (10.26 ± 1.28 g vs. 10.39 ± 1.07 g, *p* = 0.99). The weight of the SM muscle did not differ between NL and CH for the winter season (1.73 ± 0.08 g vs. 2.01 ± 0.2 g, *p* = 0.75) and for the summer season (1.27 ± 0.2 g vs. 1.76 ± 0.28 g, *p* = 0.39). Additionally, no differences in SM weight were found between winter and summer birth for NL (1.73 ± 0.08 g vs. 1.27 ± 0.2 g, *p* = 0.41) and for CH (2.01 ± 0.2 g vs. 1.76 ± 0.28 g, *p* = 0.83).

### 3.2. Longissimus Thoracis: Morphological and Histometric Evaluation—Hematoxylin–Eosin (HE) Staining

**Figure 1:**
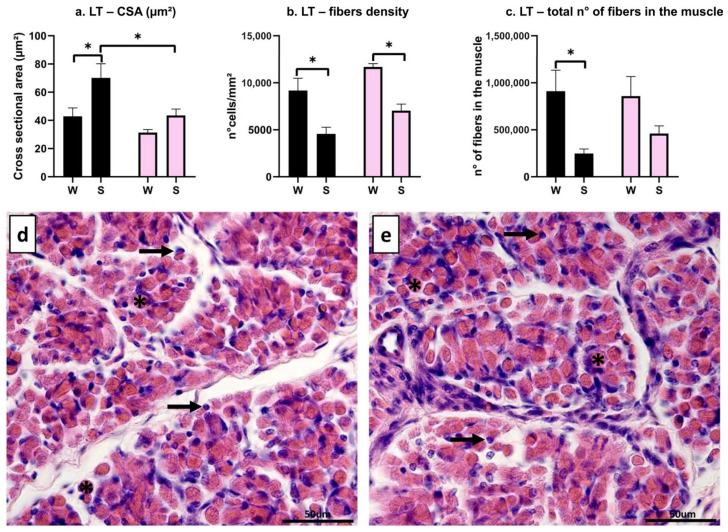
Morphometric evaluation of muscle LT. (**a**): cross-sectional area (um^2^), interaction between breed and birth season; (**b**): cell density (n° muscle fibers/mm^2^), interaction between breed and birth season; (**c**): total number of fibers, interaction between breed and birth season; (**d**): HE staining in NL/W; (**e**): HE staining in NL/S. Black bar: Nero di Lomellina (NL); pink bar: Commercial Hybrid (CH); W: animals born in winter; S: animals born in summer. Asterisk: transversal fiber; arrow: nucleus. Results are shown as mean ± SEM. * *p* < 0.05. Scale bar 50 µm.

The effect of the interaction between the breed and the birth season revealed a significant difference in transverse fibers’ CSA between NL/S and CH/S, with larger fibers in NL (*p* = 0.04); moreover, NL/S showed larger fibers than NL/W (*p* = 0.035), but no differences were observed in CH between W and S (*p* = 0.53) (Figure 1a). As for the number of muscle fibers per mm^2^, a higher muscle fiber density was found in W than in S, both in NL (*p* = 0.013) and in CH (*p* = 0.012); no differences were spotted between the breeds (W: *p* = 0.24; S: *p* = 0.26) (Figure 1b). As for the total number of fibers, NL/W showed more muscle fibers than NL/S (*p* = 0.05), while no differences were found between CH/W and CH/S (*p* = 0.35) (Figure 1c). The total number of muscle fibers between the two breeds did not differ, neither in W (*p* = 0.99) nor in S (*p* = 0.79) (Figure 1c). Representative images of Hematoxylin–Eosin staining of NL/W (Figure 1d) and NL/S (Figure 1e) for muscle LT are reported: transversal fibers appear round–polygonal (asterisks), and nuclei are located at the periphery of the cell (arrows).

### 3.3. Longissimus Thoracis: Gene Expression Evaluation—MRFs

**Figure 2:**
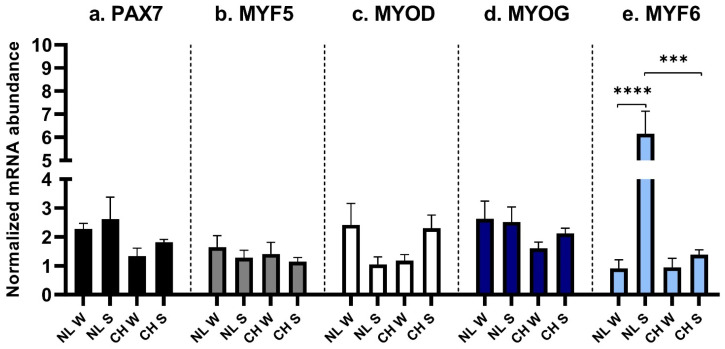
MRFs expression in LT. (**a**): *PAX7* expression, interaction between breed and birth season; (**b**): *MYF5* expression, interaction between breed and birth season; (**c**): *MYOD* expression, interaction between breed and birth season; (**d**): *MYOG* expression, interaction between breed and birth season; (**e**): *MYF6* expression, interaction between breed and birth season. NL: Nero di Lomellina; CH: Commercial Hybrid; W: animals born in winter; S: animals born in summer. Black bar: *PAX7*; gray bar: *MYF5*; white bar: *MYOD*; blue bar: *MYOG*; light blue bar: *MYF6*. *BETA ACTIN* and *DRAP1* were used for normalization. Data are shown as mean ± SEM. *** *p* < 0.001; **** *p* < 0.0001.

No significant differences were found for the expression of *PAX7* (Figure 2a), *MYF5* (Figure 2b), *MYOD* (Figure 2c), and *MYOG* (Figure 2d), neither from the interaction of the considered factors nor for the analyses of the single factors. *MYF6* was strongly expressed in NL/S fibers compared to NL/W (*p* < 0.0001) and CH/S ones (*p* = 0.0002) (Figure 2e).

### 3.4. Longissimus Thoracis: Gene Expression Evaluation—HSPs and CSPs

**Figure 3:**
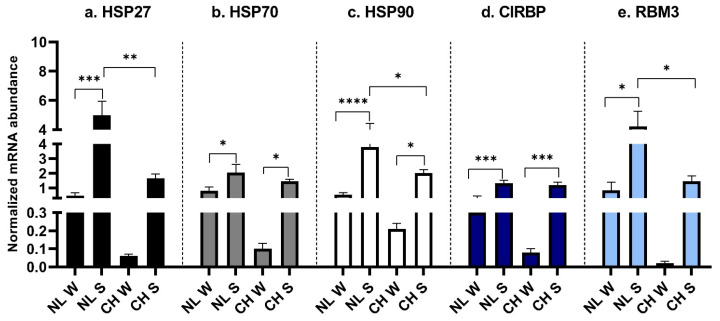
HSPs and CSPs expression in LT. (**a**): *HSP27* expression, interaction between breed and birth season; (**b**): *HSP70* expression, interaction between breed and birth season; (**c**): *HSP90* expression, interaction between breed and birth season; (**d**): *CIRBP* expression, interaction between breed and birth season; (**e**): *RBM3* expression, interaction between breed and birth season. NL: Nero di Lomellina; CH: Commercial Hybrid; W: animals born in winter; S: animals born in summer. Black bar: *HSP27*; gray bar: *HSP70*; white bar: *HSP90*; blue bar: *CIRBP*; light blue bar: *RBM3*. *BETA ACTIN* and *DRAP1* were used for normalization. Data are shown as mean ± SEM. * *p* < 0.05; ** *p* < 0.01; *** *p* < 0.001; **** *p* < 0.0001.

The expression of *HSP27* was higher in NL/S if compared to NL/W (*p* = 0.0001) and CH/S (*p* = 0.0018) (Figure 3a). *HSP70* was higher in the summer birth for both breeds (*p* = 0.044 for NL and *p* = 0.0018 for CH) (Figure 3b). *HSP90* was mainly expressed in NL/S if compared to NL/W (*p* < 0.0001) and CH/S (*p* = 0.023); moreover, a higher expression was also observed in CH/S if compared to CH/W (*p* = 0.027) (Figure 3c). The expression of *CIRBP* in LT was higher in S in both breeds (*p* = 0.003 for NL, *p* = 0.0008 for CH), but no differences were spotted between the breeds (Figure 3d). NL/S showed a higher level of *RBM3* if compared to NL/W (*p* = 0.01) and to CH/S (*p* = 0.036) (Figure 3e).

### 3.5. Semimembranosus: Morphological and Histometric Evaluation—Hematoxylin–Eosin (HE) Staining

**Figure 4:**
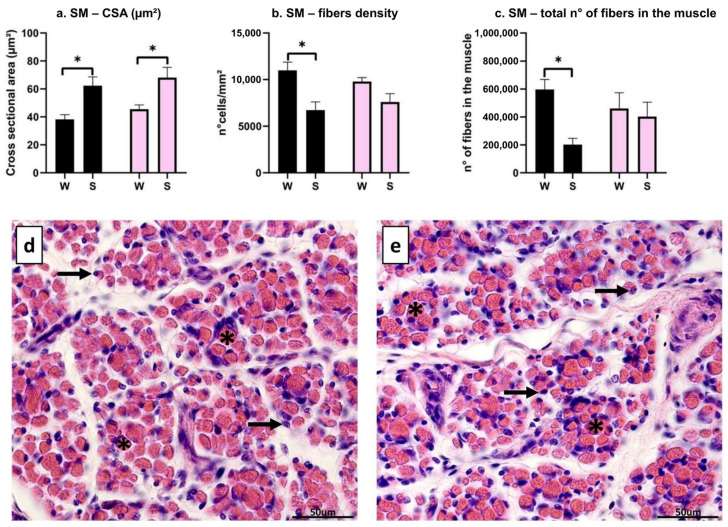
Morphometric evaluation of muscle SM. (**a**): cross-sectional area (um^2^), interaction between breed and birth season; (**b**): cell density (n° muscle fibers/mm^2^), interaction between breed and birth season; (**c**): total number of fibers, interaction between breed and birth season; (**d**): HE staining in NL/W; (**e**): HE staining in NL/S. Black bar: Nero di Lomellina (NL); pink bar: Commercial Hybrid (CH); W: animals born in winter; S: animals born in summer. Asterisk: transversal fiber; arrow: nucleus. Results are shown as mean ± SEM. * *p* < 0.05. Scale bar 50 µm.

The CSA of the transversal fibers resulted in significant differences only within each breed: in S, larger muscle fibers were found in both NL (*p* = 0.035) and CH (*p* = 0.05), but no differences were spotted between breeds (W: *p* = 0.78; S: *p* = 0.88) (Figure 4a). The number of muscle fibers per mm^2^ appeared to be higher in NL/W than in NL/S (*p* = 0.013) (Figure 4b), while no differences were found in CH (*p* = 0.28) (Figure 4b). As for the total number of fibers, NL/W showed more fibers than NL/S (*p* = 0.033); no differences were found between birth seasons for CH (*p* = 0.96), and between NL and CH, both for the winter birth (*p* = 0.71) and for the summer one (*p* = 0.41) (Figure 4c). Representative images of Hematoxylin–Eosin staining of NL/W and NL/S for muscle SM are shown in Figure 4d,e, respectively: transversal fibers are round–polygonal (asterisks), and nuclei are located at the periphery of the cell (arrows).

### 3.6. Semimembranosus: Gene Expression Evaluation—MRFs

**Figure 5:**
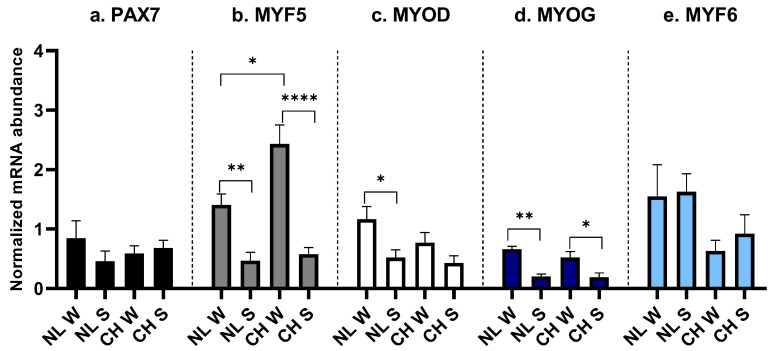
MRF expression in SM. (**a**): *PAX7* expression, interaction between breed and birth season; (**b**): MYF5 expression, interaction between breed and birth season; (**c**): *MYOD* expression, interaction between breed and birth season; (**d**): *MYOG* expression, interaction between breed and birth season; (**e**): *MYF6* expression, interaction between breed and birth season. NL: Nero di Lomellina; CH: Commercial Hybrid; W: winter birth; S: summer birth. Black bar: *PAX7*; gray bar: *MYF5*; white bar: *MYOD*; blue bar: *MYOG*; light blue bar: *MYF6*. *BETA ANCTIN* and *DRAP1* were used for normalization. Data are shown as mean ± SEM. * *p* < 0.05; ** *p* < 0.01; **** *p* < 0.0001.

No significant differences were found for the expression of *PAX7* (Figure 5a). *MYF5* was higher in W in both breeds (Figure 5b, *p* = 0.008 for NL and *p* < 0.0001 for CH). Additionally, *MYF5* was higher in CH/W if compared to NL/W (Figure 5b, *p* = 0.012). *MYOD* was higher in NL/W if compared to NL/S (Figure 5c, *p* = 0.045). *MYOG* was higher in W in both breeds (Figure 5d, *p* = 0.002 for NL and *p* = 0.025 for CH), without any difference between them. No differences between groups were found for the expression of *MYF6* (Figure 5e).

### 3.7. Semimembranosus: Gene Expression Evaluation—HSPs and CSPs

**Figure 6:**
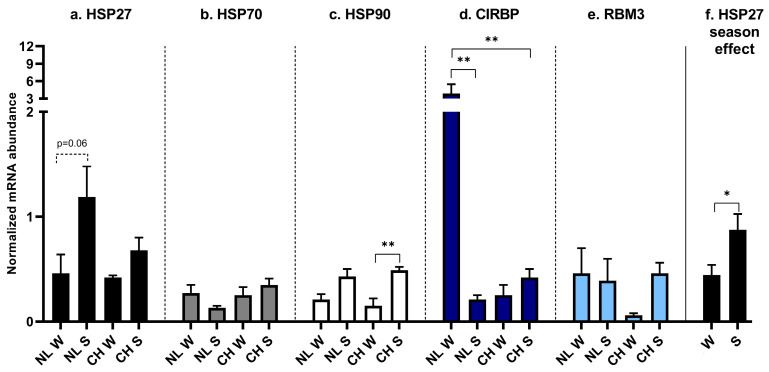
HSPs and CSPs expression in SM. (**a**): *HSP27* expression, interaction between breed and birth season; (**b**): *HSP70* expression, interaction between breed and birth season; (**c**): *HSP90* expression, interaction between breed and birth season; (**d**): *CIRBP* expression, interaction between breed and birth season; (**e**): *RBM3* expression, interaction between breed and birth season; (**f**): *HSP27* expression, effect of the birth season. NL: Nero di Lomellina; CH: Commercial Hybrid; W: winter birth; S: summer birth. Black bar: *HSP27*; gray bar: *HSP70*; white bar: *HSP90*; blue bar: *CIRBP*; light blue bar: *RBM3*. *BETA ACTIN* and *DRAP1* were used for normalization. Dashed line: tendency. Data are shown as mean ± SEM. * *p* < 0.05; ** *p* < 0.01.

The interaction of breed and season of birth revealed that *HSP27* tended to be higher in NL/S if compared to NL/W (*p* = 0.06) (Figure 6a). No significant differences were found in *HSP70* (Figure 6b). *HSP90* was higher only in CH/S if compared to CH/W (*p* = 0.004) (Figure 6c). *CIRBP* was higher in NL/W if compared to NL/S (*p* = 0.003) and CH/W (*p* = 0.004) (Figure 6d). No significant differences were found in the expression of *RBM3* (Figure 6e), neither between breeds nor between birth seasons. Birth season turned out to play a role in *HSP27* expression, with significantly higher levels in S (*p* = 0.015) (Figure 6f).

## 4. Discussion

Body growth and meat quality parameters are key objectives in pig breeding. The local pig breeds are characterized by a slower growth rate in comparison to commercial pigs, but they produce high-quality meat. However, this slower growth poses a challenge for breeders. [40]. Our recent preliminary data showed that NL growth is comparable with the hybrid commercial breed in piglets born in summer, even if a slower growth rate was observed in NL piglets born in winter compared with the commercial one [40,41]. A deeper understanding of the mechanisms driving muscle growth may provide valuable insights for optimizing breeding strategies and improving meat quality. Previous studies have mainly focused on muscle development in adult pigs, showing that rustic and local breeds generally produce higher-quality meat with distinct physicochemical and sensory traits, as reported for the British Large Black, Chato Murciano, and French Basque breeds [42,43,44,45]. However, the molecular mechanisms underlying meat characteristics still need further investigation. Studying muscle development and its influencing factors at birth could therefore offer key insights into postnatal muscle growth, potentially informing strategies to improve meat yield and quality. In this study, we tested the hypothesis that muscle development at birth varies according to breed and season. We performed a comparative analysis between NL and CH newborn piglets from the same farm, including only live-born animals without malformations or bone fractures. Animals were also homogeneous with respect to body weight, which was considered appropriate for their age. From our perspective, the choice of this model ensured appropriate experimental conditions. We focused on muscle morphology, Myogenic Regulatory Factors, and heat/cold shock proteins to provide an overview of the level of muscle development at birth across breeds and seasons. To this end, we primarily investigated muscle morphological features. NL displayed both smaller and more numerous fibers in winter in both muscles, suggesting a possible effect of seasonal conditions on muscle development. CH showed a similar pattern, but only in the SM muscle. Additionally, in summer, NL exhibited wider fibers than CH in the LT muscle, indicating a potential breed effect. According to the literature, muscle fibers in pigs are formed during fetal life, and their number is fixed before birth [46]. Postnatal muscle growth relies on hypertrophic events, which increase fiber CSA [47,48,49], and a larger CSA at birth is generally associated with more mature muscle tissue [43,49]. Postnatally, an association between muscle fiber features and productivity was found in pigs, with faster muscle growth associated with more fibers and smaller fiber diameters [50,51]. Furthermore, recent research has shown that environmental temperature affects pig muscle morphometric characteristics, with elevated temperatures associated with larger fiber CSA [52]. Overall, our findings align with the existing literature, indicating that both breed and birth season can distinctly influence muscle fibers at birth, though in different ways. These early differences may contribute to variations in muscle development, adult muscle properties, and, ultimately, meat quality, as also reported by Pallaoro et al. [34].

To better understand these morphological findings, we investigated the expression of MRFs, which regulate myogenesis. This complex process involves progenitor proliferation and simultaneous differentiation, as cells exit the cell cycle and activate molecular programs [6]. Consistent and efficient growth depends on the precise balance between progenitor proliferation and cell cycle exit [2,53]. As cells exit the cell cycle, they commit to the differentiation program, characterized by the downregulation of *PAX7* expression and the activation and completion of the MRFs cascade [6]. Therefore, a *PAX7* progenitor can follow three distinct pathways: (i) symmetric division, yielding two progenitor cells; (ii) asymmetric division, producing one progenitor cell and one differentiating cell; (iii) terminal division, resulting in two differentiating cells [54]. Since we did not observe any differences in *PAX7* expression for both muscles, breeds, and birth season, we may hypothesize that a terminal division is occurring, so that the expression of satellite cells cannot be properly quantified. During myogenesis, MRFs are expressed in a time-specific manner [10]. The early factors MYF5 and MYOD guarantee myoblast proliferation and commitment, while the late factors MYOG and MYF6 promote myotube formation and muscle fiber maturation [6,10,11]. Gao et al. [55] demonstrated that LT cells from local Chinese Lantang piglets differentiate faster than those from Landrace pigs in vitro, forming wider myotubes and exhibiting higher MRF expression, which suggests an effect of breed on myogenesis. In vivo, the opposite pattern was observed: commercial breeds tend to exhibit faster myogenesis and more developed muscles than native breeds, likely due to the lower muscle mass of the latter. [56]. Our analysis of MRFs revealed significant differences between the two breeds and muscles, supporting our morphological observations. LT showed higher MYF6 expression in summer-born NL, which correlates with the larger fibers’ CSA observed morphologically: indeed, MYF6 is responsible for the terminal differentiation of muscle fibers and is generally associated with larger muscle fiber size [57]. SM in winter appeared in a transitional phase between proliferation and differentiation, as indicated by high MYF5, MYOD, and MYOG expression, which aligns with the smaller fibers observed in both breeds during this season. Considering that LT is a positional muscle and SM is a locomotor muscle [58], we hypothesize that the observed differences reflect their distinct functions. As previously demonstrated, these two muscles strongly differ in gene expression and protein profile, which also explains differences in the pork products [31,59]. Our study suggests that LT and SM at birth show distinct muscle development, which are differently impacted by the breed and the season of birth. This may also support the differences documented in LT and SM from adult pigs [31]. However, further studies are needed to better understand these differences and their implications for pork quality.

Given the observed differences between breeds and birth seasons, we hypothesize that distinct stressors may have led to varying levels of stress-related proteins across the groups, potentially influencing muscle development. We therefore focused on HSPs expression. According to literature, the increased expression of HSPs is crucial for adapting to various stressors, including thermal, oxidative, and mechanical challenges [60,61,62]. Consequently, some authors suggest that the capacity to express these chaperone proteins is closely linked to the animal’s resilience, which is generally higher in local and native breeds [63]. Most literature concerning HSPs’ implication in the myogenic process refers to in vitro studies. Metzger et al. [22,23] demonstrated that pig satellite cells under heat stress conditions accelerate their development by increasing and enhancing myotube formation, alongside an upregulation of HSPs. Similarly, Harding et al. [64] found that chicken satellite cells from both anaerobic pectoralis major and aerobic biceps femoris showed enhanced myogenic activity at higher temperatures and reduced activity under hypothermic conditions. In our study, both LT and SM muscles of summer-born piglets showed higher HSP expression, likely correlating with the observed myogenic features. Interestingly, only LT exhibited higher expression of all these chaperone proteins in the local NL breed, consistent with our previous observations. However, further studies are required to better elucidate the underlying causes of the variations in HSP expression across seasons in vivo so as to better identify potential stressors and HSP inducers. Unlike HSPs, the role of CSPs in mammals’ muscles remains unclear, since these molecules are released under various stress conditions, not just thermal ones [65]. In brown bears, CSP expression increases during the hibernation period to counteract the induced oxidative stress, suggesting that these proteins could be involved in preventing muscle atrophy [66]. In our study, CSP expression differed according to muscle and season. CIRBP was higher in LT in summer and in SM in winter, whereas RBM3 was upregulated in summer, with the highest levels in NL, suggesting an activation process in the local breed. Further studies are required to better understand the differential expression of CSPs and their role in muscle development, particularly under stress conditions. A limitation of this study, in fact, could be the targeted nature of the molecular analysis, which was focused on a limited gene panel that may not reflect broader transcriptional changes. Further studies employing more comprehensive transcriptomic approaches could help to better characterize the molecular pathways involved and further support the present findings.

## 5. Conclusions

Our study demonstrated that the level of muscle development at birth in pigs can be influenced by both breed and birth season. While birth season influenced both LT and SM, the genetic background had a more pronounced effect on the LT muscle. The differences between these muscles are likely to reflect the distinct muscle functions, which also have implications for meat quality. Our study confirmed that some differences are already present at birth and suggests that environmental conditions could shape early muscle development. However, further research is needed to better elucidate the molecular mechanisms underlying the modulation of myogenic potential by breed and birth season, its evolution throughout the animal’s life, and its impact on the quality of pork.

## Figures and Tables

**Table 1:** Primers used in qPCR for the analysis of the considered targets. PF: Primer Forward; PR: Primer Reverse; bp: base pair of the amplicon size; Accession No.: accession number of the mRNA sequence; Ref.: reference for the primer sequences.

Target	PF 5′ >> 3′	PR 5′ >> 3′	bp	Accession No.	Ref.
*DRAP1*	AAGACCATGACCACATCCCACC	TTTGCTTCCCATGCCACCGTTC	157	XM_003468252.2	[36]
*B-ACTIN*	GACATCCGCAAGGACCTCTA	ACACGGAGTACTTGCGCTCT	157	XM_003124280.5	[17]
*PAX7*	GGGGTCTTCATCAATGGGCG	GAACATGCCTGGGTTCTCCC	198	XM_021095460.1	[37]
*MYOD*	TGCAAACGCAAGACCACTAA	GCTGATTCGGGTTGCTAGAC	127	NM_001002824.1	[17]
*MYOG*	GAAAACTACCTGCCCGTCCA	CCACAGACACGGACTTCCTC	184	NM_001012406.1	[37]
*MYF5*	CCTGAATGCAACAGCCCT	CGGAGTTGCTGATCCGAT	151	NM_001278775.1	[37]
*MYF6*	ATCTTGAGGGTGCGGATTTC	CAATGTTTGTCCCTCCTTCCT	108	NM_001244672.1	[17]
*HSP27*	AGGAGCGGCAGGATGAG	GGACAGGGAGGAGGAGAC	101	NM_001007518.1	[38]
*HSP70*	GTGGCTCTACCCGCATCCC	GCACAGCAGCACCATAGGC	114	NM_001123127.1	[38]
*HSP90*	CGCTGAGAAAGTGACCGTTATC	ACCTTTGTTCCACGACCCATAG	126	U94395.1	[38]
*CIRBP*	AGGGCTGAGCTTTGACACCA	GCCCATCCACAGACTTCCCGTTC	191	NM_001246197.1	[39]
*RBM3*	TAGCCGTGTCTTCTGCCTTT	AGCCTGCTCATCTGTGTTGA	144	NM_001243419.1	[39]

## Data Availability

Results are contained within the article. The datasets used and/or analyzed during the current study are available from the corresponding author on reasonable request.

## Abbreviations

The following abbreviations are used in this manuscript:

NL	Nero di Lomellina
CH	Commercial Hybrid
W	Winter
S	Summer
LT	*Longissimus thoracis*
SM	Semimembranosus
MRFs	Myogenic Regulatory Factors
HSPs	Heat shock proteins
CSPs	Cold shock proteins
